# Berberine alleviates ischemia reperfusion injury induced AKI by regulation of intestinal microbiota and reducing intestinal inflammation

**DOI:** 10.1186/s12906-023-04323-y

**Published:** 2024-01-30

**Authors:** Aijing Huo, Fengmei Wang

**Affiliations:** 1 Department of Nephropathy and Immunology, The Third Central Clinical College of Tianjin Medical University, Tianjin, China; 2 Tianjin Key Laboratory of Extracorporeal Life Support for Critical Diseases, Artificial Cell Engineering Technology Research Center, Tianjin Institute of Hepatobiliary Disease, The Third Central Hospital of Tianjin, Tianjin, China; 3 Department of Gastroenterology and Hepatology, The Third Central Clinical College of Tianjin Medical University, Tianjin, China

**Keywords:** Berberine, Intestinal barrier, Acute kidney injury, Intestinal microbiota

## Abstract

**Background:**

It has been found that a variety of host disease states can exacerbate intestinal inflammation, leading to disruption of intestinal barrier function. Changes in the composition of the intestine microbiota, which affect downstream metabolites in turn, ultimately react against the host.

**Objectives:**

We revealed the mechanism of berberine as an intestinal protective agent in rats with renal ischemia–reperfusion injury acute kidney injury (AKI).

**Methods:**

HE staining was performed to evaluate the pathological changes in the colon and kidney. 16 S rRNA analysis was performed to assess the intestinal microbiota. Intestine TLR4/NF-κB expression was assessed by western blot. Q-RT-PCR was performed to detect TLR4 in intestine and IL-6 and KIM-1 gene expression in the kidney. SPSS 22.0 was used to compare the data.

**Results:**

Rats with AKI exhibited increased relative abundances of *Proteobacteria* and *Bacteroidetes* and decreased relative abundances of *Lactobacillus*, *Ruminococcus* and *Lachnospiraceae* belonging to the phylum *Firmicutes*. The Sirt1-NF-κB-TLR4 pathway was involved in the occurrence process, accompanied by intestinal inflammation and oxidation. Berberine reversed the appeal change.

**Conclusion:**

Berberine inhibits the intestinal biological barrier of *Proteobacteria*, reduces LPS production, exerts an anti-inflammatory effect, and delays the progression of AKI.

**Supplementary Information:**

The online version contains supplementary material available at 10.1186/s12906-023-04323-y.

## Introduction

The intestinal barrier and microbiota regulate the biological and immunological functions of the intestinal together [1]. The individual’s inflammatory status is influenced by the crosstalk between their intestinal microbiota and the host, which is related to changes in the host’s diets and physiological state [2]. The structure and function of the intestinal barrier is required to maintain healthy intestinal metabolic function. And intestinal permeability is also a measure of altered intestinal barrier function in humans [3]. Previous studies have reported that the interaction between the liver–intestinal and brain–intestinal axes affect the progression and outcome of related diseases [4, 5].

Acute kidney injury (AKI) is characterized by a dramatic decline in kidney function and has high mortality and morbidity, threatening millions of patients worldwide [6]. The KDIGO guidelines emphasize early management of AKI treatment, particularly the initial stage of AKI development, such as the suspected AKI stage. Acute tubular necrosis is the main cause of AKI. There have also been some previous studies on the renal-intestinal axis. A recent study reported that the “sterile” intervention of antibiotics on the intestinal microbiota in rats with AKI can improve renal function [7].

Berberine, a naturally occurring plant alkaloid obtained from barberry and Coptis chinensis, is used to cure diarrhea in China. It has been discovered that intestinal microbiota plays a significant role in mediating the pharmacokinetics and biological effects of berberine [8]. Berberine can inhibit the growth of harmful intestinal bacteria and enhance the population of beneficial bacteria such as *Bifidobacterium adolescentis* and *L. acidophilus*. The dosage of berberine in animal experiments ranges from 50 to 300 mg/kg/d, and it is safe to use with no obvious adverse reactions [9, 10].

Several recent studies have reported the interaction of berberine, Sirt1, oxidative stress, and inflammatory responses [11, 12]. Sirt1 is a conserved mammalian NAD-dependent protein deacetylase. Sirt1 suppresses NF-κB signaling and as a result reduces NF-κB driven inflammation greatly [13]. It is critical to explore whether the Sirt1/NF-κB signalling pathway plays a regulatory role in oxidative stress and inflammation in AKI induced intestine.

In this study, we mainly aimed to assess the important roles of the intestinal biological and physical barriers in an AKI model, we chose 150 mg/kg/d and also obtained expected experimental results.

## Materials and methods

### Experimental models and laboratory animals

Male Sprague-Dawley (SD) rats (SPF grade, 230-280 g, 6-8-week old; Beijing Huafukang) were raised in the animal lab of Tianjin Nankai Hospital at 20-25 °C with free access to food and water and regular 12:12 h indoor light changes.

**Fig. 1:**
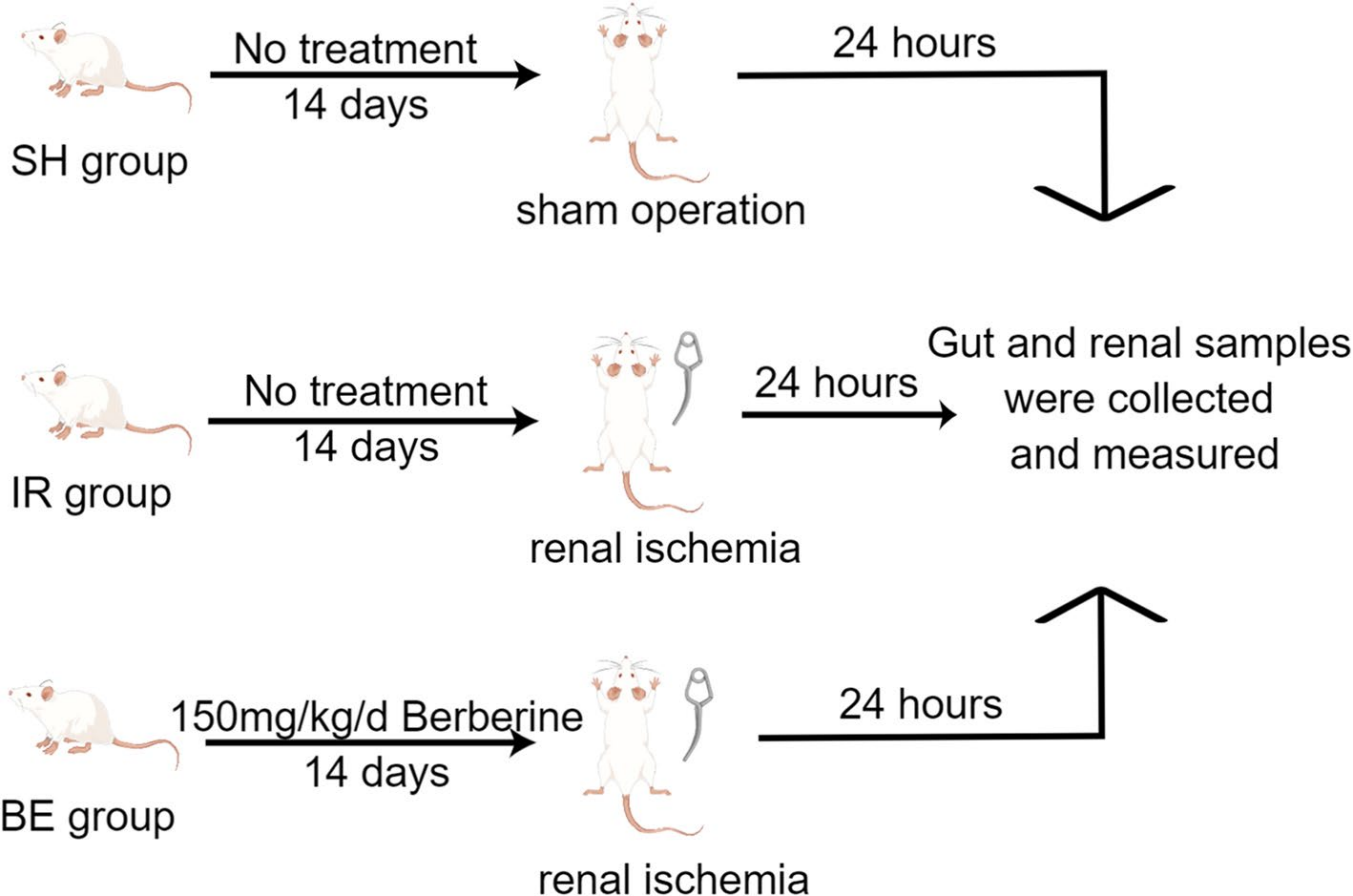
Experimental design for assessing berberine’s effect on AKI rats (*n* = 6/group) (Drawn by Figdraw). SH: sham-operated group, IR: AKI group, BE: berberine-pretreated AKI group

In total, 18 SD rats were randomly allocated into the following three groups (Fig. 1): (1) The sham-operated group (SH, *n* = 6): The rats underwent same surgical procedures but without renal artery clipping. (2) The ischemia–reperfusion damage group (IR, *n* = 6). The rats were anesthetized by intraperitoneal injection of sodium pentobarbital (starting with 50 mg/kg and supplemented with 5 mg/kg during the surgery if necessary), and the surgery was performed after the shallow reflex disappeared. Bilateral renal ischemia-reperfusion (IR) injury model was performed as previously described with slight modifications [14]. For the induction of ischemia–reperfusion injury (IRI), after kidney externalization, the renal pedicle was clamped for 60 min, then the clamp was released to allow blood to reperfuse the kidney. The reperfusion time was set to 24 h. (3) The berberine intervention group (BE group, *n* = 6): The rats were gavaged with 150 mg/kg/d of berberine (Macklin, B832574) for 14 days. The berberine was dissolved in drinking water for rat. Clinical symptoms, such as unmitigable severe pain and incapable of maintaining normal activities or eating on their own, were humane endpoints used in order to determine when the animals should be sacrificed to minimize suffering [15].

The rats were given CO_2_ anesthetic at 24 h after IRI, blood was taken during CO2 anesthesia and colon, kidney, and colon intestinal contents were taken after euthanasia. The use of animals in this study was approved by the Tianjin Nankai Hospital’s Animal Care and Ethics Committee (SYXK2020-0008).

### Evaluation of the colon and kidney tissues

Reference to previous methods, hematoxylin and eosin (HE) were used to stain the colon and kidney tissues [16]. The inflammation of colon tissue was evaluated according to the degree of infiltration of inflammatory cells. Kidney tissue damage was assessed by brush border loss, tubular dilation, cast formation, and cell lysis. The following scoring criteria were used to score visible damage to the renal tubules: 0 (no damage), 1 (damage < 25%), 2 (damage = 25–50%), 3 (damage = 50–75%), and 4 (damage > 75%). In each group, 10 high-power fields (⨯400) were used to evaluate the score.

### Intestinal permeability assay

FITC-dextran (4 kDa; Sigma), a nonmetabolic macromolecule, was used as intestinal permeability probe. FITC-dextran was gavaged in 20 h after IRI surgery. A single dose of FITC-dextran was 200 µL (100 mg/ml). After the rats were sacrificed, the fluorescence intensity of serum samples was measured (excitation wavelength 492 nm/emission wavelength 520 nm) [17]. The preceding operations must be performed in a dark environment.

### Immunofluorescence staining to detect the expression of Sirt1 in ileal paraffin sections

Immunofluorescence staining was performed as previously described [16, 18]. Briefly, after the section was repaired, the slices were incubated with 5% BSA at room temperature for 30 min. The slices were incubated at 4 °C overnight with the following primary antibodies: Sirt1 antibody (1:100, bs-0921R, Bioss). Then, the slices were incubated with the appropriate fluorescence-conjugated secondary antibody (1: 500, Abcam, MA, USA) at 37° C for 1 h.

### Intestinal total SOD activity assay

A total SOD activity detection kit (WST-8 method) (Beyotime, S0101S) was used to measure total SOD activity. As directed by the manufacturer, hypoxanthine and xanthine oxidase produced superoxide radicals. A total of 20 µg protein sample was mixed with 160 µL WST-8 enzyme activity mix solution (containing 151 µL SOD assay buffer, 8 µL WST-8, and 1 µL enzyme solution) and 20 µL reaction start working solution (diluted with 39 µL of SOD detection buffer for every 1 µL of reaction starter) and incubated at 37 °C for 30 min. The absorbance of samples was determined at 450 nm.

### Serum creatinine and LPS assay

The Cr level was analyzed using the automatic biochemical analyzer and averaged to reduce experimental error. According to the method described by Kim et al. [19], the LPS content in the blood was determined using diazo-conjugated Limulus reagent (LAL).

### Western blot

In Nonidet-P40 (NP40) buffer, frozen samples of tissues were homogenized and crushed. In a microcentrifuge, the protein extracts were separated by centrifugation at 16,000 g for 20 min at 4 °C. Protein was loaded into SDSPAGE gel wells in equal amounts. The protein was transferred from the gel to the membrane. Membranes were probed overnight at 4 °C with primary antibody: NF-κB-p65 (dilution 1:100, CST, 8242), p- NF-κB-p65 (dilution 1:100, CST, 3033), TNF-α (dilution 1:100, Abcam, Ab6671, Danvers, MA, USA), β-actin (dilution 1:100, CST, 3700), secondary antibody Anti-rabbit IgG, HRP-linked Antibody (dilution 1:100, CST, 7074) incubation.

### Real-time quantitative PCR

Real-time PCR was used to evaluate the mRNA expression of TNF-α, IL-6, IL-1β, TLR4 and Sirt1 in the colon and IL-6 and KIM-1 in the kidney. RNA was collected using the RNeasy Mini kit (Qiagen, CA). After the concentration was determined, the RNA was reverse-transcribed according to the manufacturer’s protocol. The cDNA template was amplified with primers of the sequences shown in Table 1 according to the manufacturer’s protocol (Life Technologies Corporation, Chongqing, China).

**Table 1:** List of RT-PCR primer sequences

target gene	RT‒PCR primer sequences
IL-6-RAT-qF IL-6-RAT-qR	TCTCTCCGCAAGAGACTTCC CTGGTCTGTTGTGGGTGGTA
IL-1β-RAT-qF IL-1β-RAT-qR	AGGAGAGACAAGCAACGACA TTGGGATCCACACTCTCCAG
KIM-1-RAT-qF KIM-1-RAT-qR	CAAGACCCACAACCACAAGG CCATTCCAGTCTGCAGGAGTA
SIRT1-RAT-qF SIRT1-RAT-qR	AGCGTCTTGACGGTAATCAA AACTTGGACTCTGGCATGTG
TNF-α-RAT-qF TNF-α-RAT-qR	CGTCGTAGCAAACCACCAAG CCCTTGAAGAGAACCTGGGA
TLR4-RAT-qF TLR4-RAT-qR	TAGCCATTGCTGCCAACATC CCTCAGCAAGGACTTCTCCA
GAPDH-RAT-qF GAPDH-RAT-qR	CAAGGCTGAGAATGGGAAGC GAAGACGCCAGTAGACTCCA

### Analysis of the intestinal microbiota

A Power SoIL DNA Isolation Kit (MOBIO Laboratories) was used to extract total bacterial DNA from the samples, and primers were utilized to amplify the V3-V4 region of the bacterial 16 S rRNA gene.

- Forward primer, 5’-ACTCCTACGGGAGGCAGCA-3’;
- Reverse primer, 5’-GGACTACHVGGGTWTCTAAT-3’.

The PCR amplification was performed in a total volume of 50 µL containing 10 µL buffer, 0.2 µL Q5 High Fidelity DNA Polymerase, 10 µL High GC Enhancer, 1 µL dNTPs, 10 µM each primer and 60 ng genomic DNA.

The PCR cycle system was as follows: initial denaturation at 95 °C for 5 min, denaturation at 95 °C for 1 min, annealing at 50 °C for 1 min, 72 °C for 1 min, and extension at 72 °C for 7 min for 15 cycles. The PCR product of the first-step PCR was purified by VAHTS™ DNA Clean Beads. A second round of PCR was then performed in a 40 µL reaction containing 20 µL of 2x Phusion HF MM, 8 µL of ddH_2_O, 10 µM of each primer and 10 µL of PCR product from the first step. The cycle system was as follows: initial denaturation at 98 °C for 30 s, 98 °C for 10 s, 65 °C for 30 s, 72 °C for 30 s, and extension at 72 °C for 5 min for 10 cycles. Finally, all PCR products were quantified by Quant-iT™ dsDNA HS Reagent and pooled together.

### Data processing and analysis

SPSS 22.0 software was used for statistical analysis. Analysis of variance (ANOVA) was performed to compare the differences among the groups. The independent sample *t*-test or the Mann–Whitney *U* test were used to compare two continuous variables. *P* value ≤ 0.05 was considered significant. The Shannon and Simpson indices of each sample at the 97% similarity level were calculated to analyze the species diversity within a single sample; the variations in species diversity (community composition and structure) of different samples were compared using diversity analysis. According to the distance matrix, the PCA of the samples at the appropriate distance was computed. Through the significance of variations between the groups, biomarkers with substantial differences were identified between various groups. 16 S rRNA functional gene prediction analysis was used to predict the gene function of the samples and calculate the functional gene abundance.

## Results

### Rats with AKI exhibited increased intestinal inflammation and promoted apoptosis, and berberine attenuated AKI-induced intestinal inflammation

**Fig. 2:**
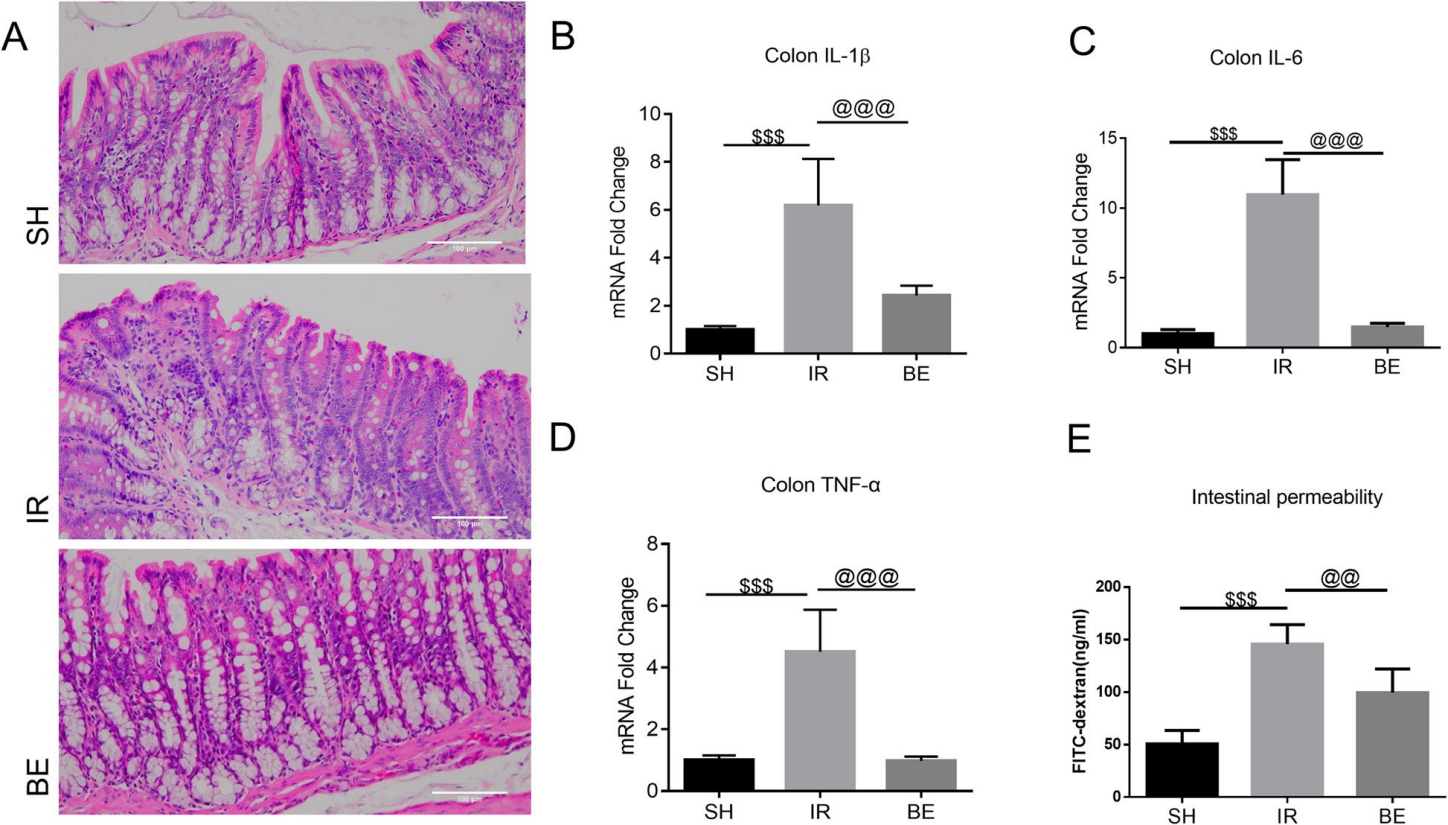
AKI rats increased intestinal inflammation and promoted apoptosis, and berberine attenuated AKI-triggered intestinal inflammatory responses. **A** Representative HE-stained colon tissue pictures (⨯200). Q-RT-PCR was used to assess inflammatory relative mRNA expression of **B** IL-1β, **C** IL-6, and **D** TNF-α in colon. When compared to the SH group, all data were log2-transformed and reported as fold changes in expression levels (mean value of SH group was set to 1). IL-1β:  IR vs. SH, ^$^*P* <0.001, IR vs. BE, ^@^*P* <0.001; IL-6:  IR vs. SH, ^$^*P* <0.001, IR vs. BE, ^@^*P* <0.001; TNF-α: IR vs. SH, ^$^*P* <0.001, IR vs. BE, ^@^*P* <0.001. **E** Serum FITC-dextran assay reflected the intestinal permeability of each group. IR vs. SH, ^$^*P* <0.001, IR vs. BE, ^@^*P* <0.01; data were presented as the mean ± SD, *n* = 6, and analyzed using one-way ANOVA. SH: sham-operated group, IR: AKI group, BE: berberine-pretreated AKI group

The rats in the SH and IR groups did not receive berberine (Fig. 1), whereas those in the BE experimental group received 150 mg/kg/day of berberine for 14 consecutive days. The frequency of neutrophils, lymphocytes, and polymorphonuclear leukocytes in the colonic lamina propria of rats with AKI was higher than that of the SH group, according to HE staining of rat colon tissues (Fig. 2A). Rats with AKI exhibited considerably increased gene levels of the colonic inflammatory factors such as IL-1β, IL-6, and TNF-α compared with those in the SH group (Fig. 2B–D). In contrast, berberine inhibited the transcription of intestinal-related inflammatory genes. AKI rat serum exhibited higher levels of FITC-dextran, and intestinal permeability was improved in the berberine group compared with that in rats with AKI (*P* < 0.05) (Fig. 2E). These findings indicated that berberine appeared to attenuate AKI-induced intestinal inflammation and restore intestinal injury in rats. Finally, berberine reduced AKI-induced intestinal inflammation via modulating the expression of intestinal-inflammation-related genes.

### The intestinal microbiota was disrupted in AKI rats, and berberine affected the composition of the intestinal microbiota

**Fig. 3:**
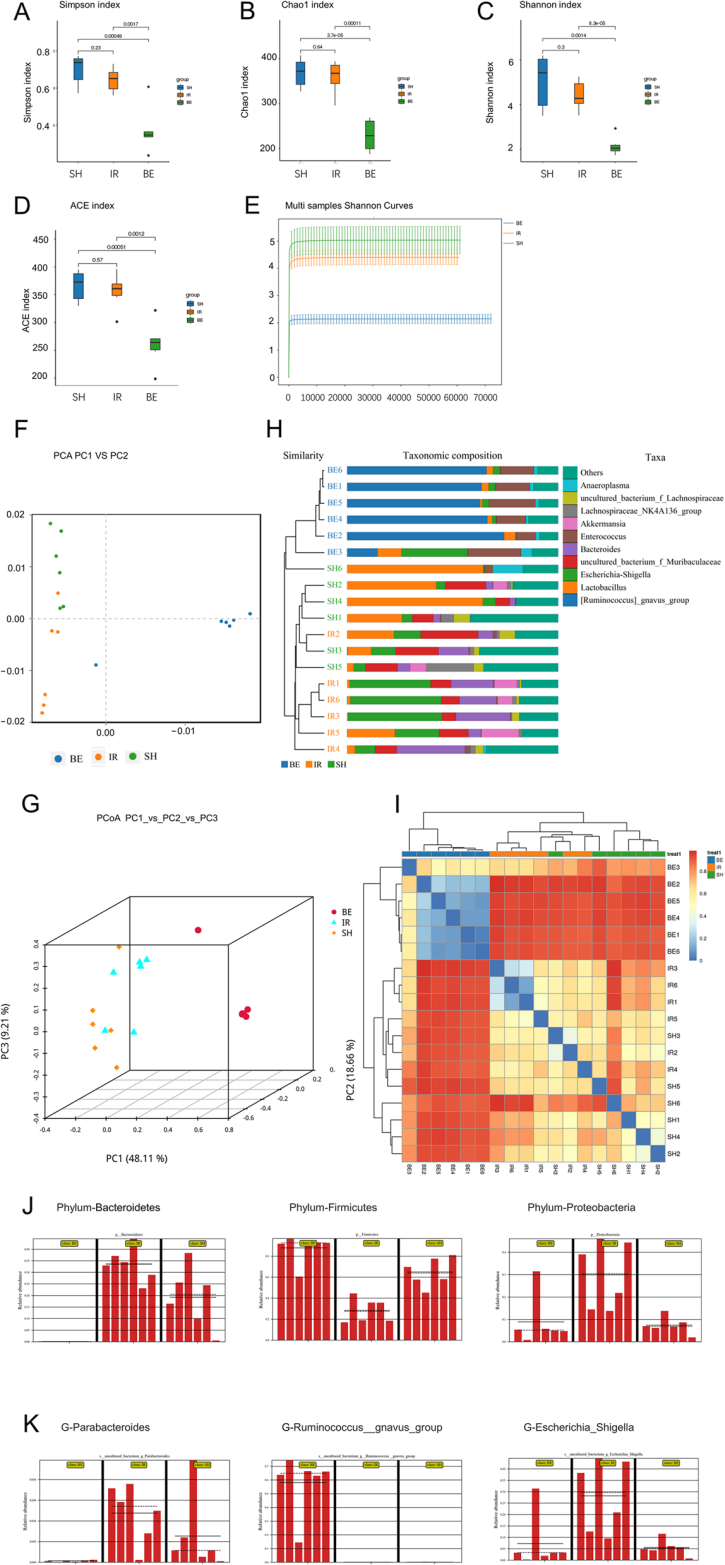
Intestinal microbiota was disturbed in AKI rats, and berberine altered the composition of the intestinal microbiota. Alpha diversity was traced with the **A** Simpson index, **B** Chao1 index, **C** Shannon index, and **D** ACE index. **E** Multis-sample Shannon curves showing differences between groups. β-diversity was displayed using **F** PCA (PC1 vs. PC2), **G** PCoA (PC1_vs_PC2_vs_PC3), **H** UPGMA clustering tree combined with histogram plotting, **I** heatmap showing the relative abundance of species between groups. Main differences in species between groups were shown at the **J** species phylum level and **K** genus level. Student’s t test was employed for pairwise comparisons of alpha diversity, *n* = 6, and one-way ANOVA was utilized for analysis, data were presented as the mean ± SD, *n* = 6. SH: sham-operated group, IR: AKI group, BE: berberine-pretreated AKI group

The number and composition of the microbiota in cecal contents was investigated. Through the 16 S rRNA gene, we studied the alterations in the intestinal microbiota after berberine therapy in rats with AKI. From 18 sequenced samples, 1,439,914 pairs of reads were collected, and 1,433,561 clean reads were created after double-ended read quality control and splicing. Each sample produced at least 79,199 clean reads, with an average of 79,642 clean reads produced. The Simpson, Chao1, Shannon, and ACE indices were used to evaluate the diversity and richness of species among each group. The rats with AKI exhibited both the microbial diversity and richness decrease (Fig. 3A–E). As a natural antibacterial agent, berberine downregulated the diversity and richness of the intestinal microbiota in rats with AKI [20, 21]. PCA (Fig. 3F), PCoA (Fig. 3G), and UPGMA clustering tree and columnar combination plot (Fig. 3H) were used to compare the degree of similarity in species diversity among the three groups. The heatmap showed the relative abundance of species between groups in Fig. 3I. The clade diagram (Fig. 3J–K) revealed the main differences between the groups in terms of intestinal species at the phylum and genus levels. The abundance of *Escherichia*, *Shigella*, and *Parabacteroides* was increased in AKI. Berberine treatment reversed the abundance of *Proteobacteria* in the intestinal microbiota in AKI group, while *Firmicutes* generating SCFAs were the dominating species.

**Fig. 4:**
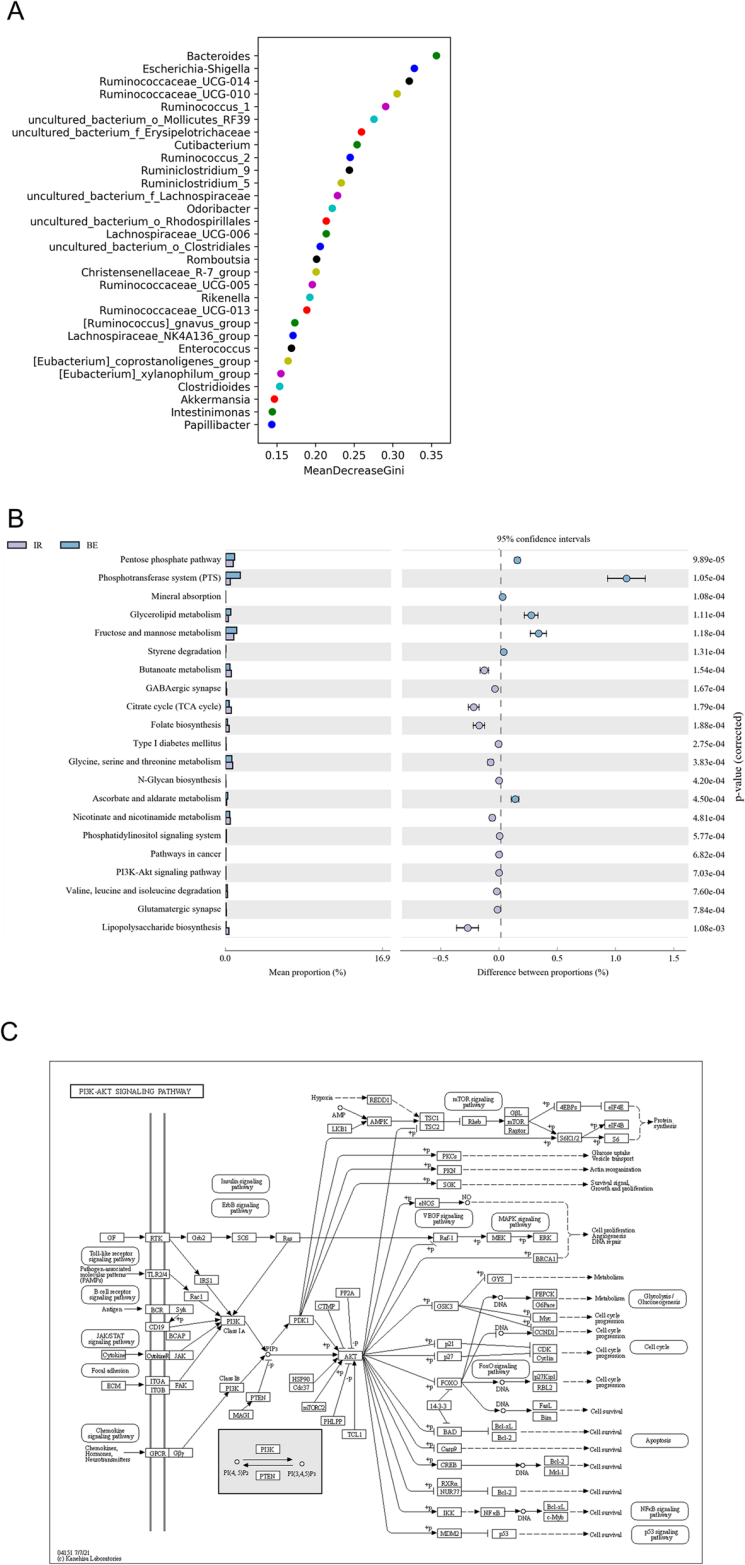
**A** Dendritic plot of LefSe analysis results. **B** Random forest plots showing major differences among the three groups at the species phylum level. **C** Adoption Picrust2 software analyzed the functional difference bar chart between BE and IR groups through the difference in KEGG metabolic pathways

Next, we used a random forest plot to screen the differential expression of species at the genus level in the three groups (Fig. 4A). The 16 S rRNA sequencing results were compared using the Picrust2 software to determine the species present in the samples. The functional gene composition was analyzed to assess the functional differences between the IR and BE groups (Fig. 4B, C). *Bacteroides*, *Escherichia* and *Shigella*, *Ruminococcaceae_UCG-014* and *Ruminococcaceae_UCG-010* may be the main altered microbiota among the three groups at the genus level. Regarding the effect of berberine on the intestinal microbiota, it was speculated that the functional genes may be involved in related pathways such as the phosphotransferase system (PTS), PI3K-Akt signaling pathway, butanoate metabolism, and lipopolysaccharide biosynthesis in KEGG [22].

In this study, differences were observed in the intestinal microbiota between AKI and berberine group. It indicated the possible mechanism of berberine on AKI rats. Firstly, the mechanism was due to a decrease in the related microbiota that produces LPS. Secondly, it was due to an increase in the related microbiota that produces SCFAs. Therefore, it was speculated that the functional genes involved in the action of berberine on intestinal microbiota were involved in intestinal inflammation and butyric acid metabolism.

### Berberine alleviates renal inflammation and improves renal damage in rats with AKI

**Fig. 5:**
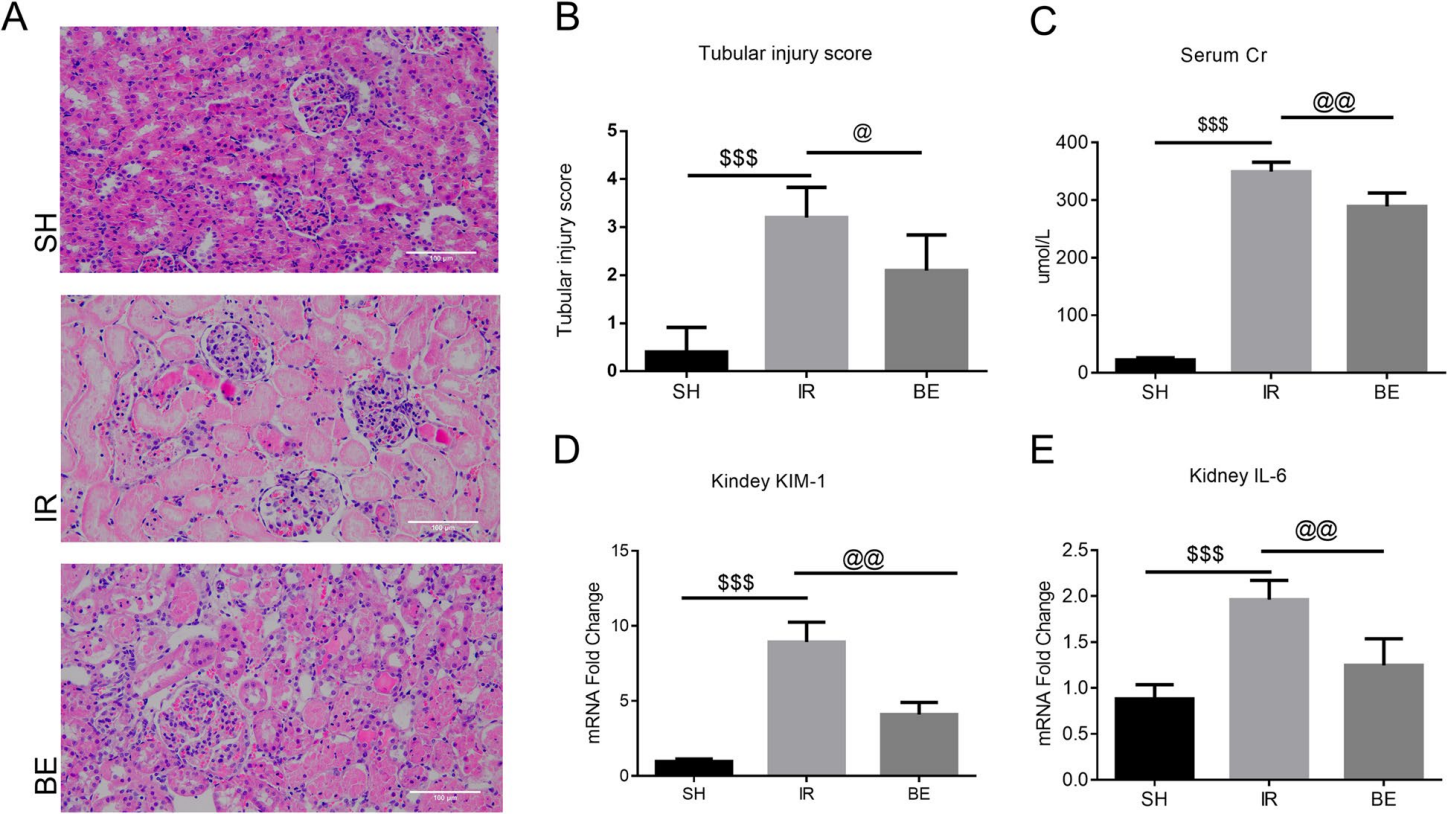
In AKI rats, berberine reduces renal inflammation and exacerbates renal damage. **A** **B** Kidney HE staining (×200), renal tubular injury score, IR vs. SH, ^$^*P* < 0.001, IR vs. BE, ^@^*P* < 0.05. **C** Serum creatinine in each group, IR vs. SH, ^$^*P* < 0.001, IR vs. BE, ^@^*P* < 0.01. **D** KIM-1 content in kidney measured by Q-RT-PCR, IR vs. SH, ^$^*P* < 0.001, IR vs. BE, ^@^*P* < 0.01. **E** The content of IL-6 in the kidney was measured by Q-RT-PCR, IR vs. SH, ^$^*P* < 0.001, IR vs. BE, ^@^*P* < 0.01. When compared to the SH group, all data were log2-transformed and reported as fold changes in expression levels (mean value of SH group was set to 1). Data were presented as the mean ± SD, *n* = 6, and analyzed using one-way ANOVA. SH: sham operation group, IR: AKI group, BE: berberine-pretreated AKI group

Renal injury was assessed through renal tubular injury score, the levels of rat serum creatinine, renal KIM-1, and IL-6 (renal inflammatory marker) gene expression. The serum creatinine and renal KIM-1 levels were markedly elevated as a result of renal tubular damage by IRI. However, berberine supplementation significantly (*P* < 0.05) reversed this trend (Fig. 5A–D). The gene expression level of IL-6 was dramatically elevated by ischemia–reperfusion injury, and berberine pretreatment significantly reduced renal inflammation (*P* < 0.01) (Fig. 5E). In summary, berberine reduced IRI-induced kidney damage.

### Berberine was involved in the colonic Sirt1-NF-κB-TLR4 inflammatory pathway

**Fig. 6:**
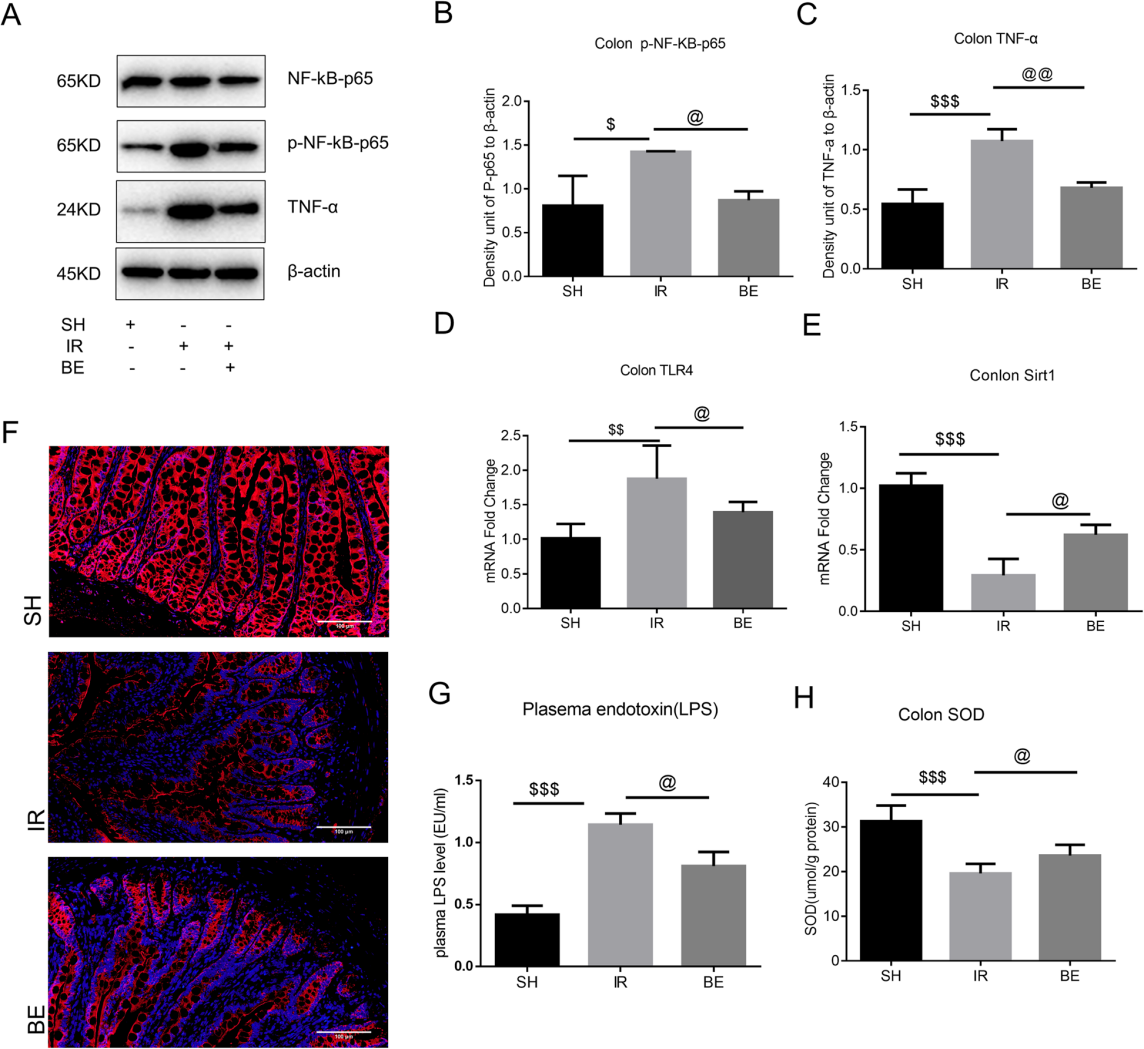
Berberine was involved in the colon Sirt1-NF-κB-TLR4 inflammatory pathway. **A** NF-κB-p65, p-NF-κB-p65 and TNF-α protein bands. **B** Histograms showing the relative intensities of p-NF-κB-p65, these bands were normalized to β-actin bands. IR vs. SH, ^$^*P* < 0.05, IR vs. BE, ^@^*P* < 0.05; (Figure supplement 1. Unedited and labeled Western blot in Fig. 5A). **C** Histograms showing the relative intensities of TNF- α, these bands were normalized to β-actin bands: IR vs. SH, ^$^*P* < 0.001, IR vs. BE, ^@^*P* < 0.01; **D** TLR4 content in colon tissues measured by Q-RT-PCR, IR vs. SH, ^$^*P* < 0.001, IR vs. BE, ^@^*P* < 0.05. When compared to the SH group, all data were log2-transformed and reported as fold changes in expression levels (mean value of SH group was set to 1). **E** Typical images of Sirt1 expression in colon tissues of each group detected by immunofluorescence. **F** Sirt1 content in colon tissues measured by Q-RT-PCR, IR vs. SH, ^$^*P* < 0.001, IR vs. BE, ^@^*P* < 0.05. When compared to the SH group, all data were log2-transformed and reported as fold changes in expression levels (mean value of SH group was set to 1). **G** LPS determined by Limulus assay, IR vs. SH, ^$^*P* < 0.001, IR vs. BE, ^@^*P* < 0.05. **H** Colonic SOD was detected using the SOD kit, IR vs. SH, ^$^*P* < 0.001, IR vs. BE, ^@^*P* < 0.05. Data was presented as the mean ± SD, *n* = 3–6, and analyzed using one-way ANOVA. SH: sham-operated group, IR: AKI group, BE: berberine-pretreated AKI group

It revealed that berberine pretreatment suppressed the AKI induced production of p-NF-κB-p65 and TNF-α (Fig. 6A-C) protein expression and TL-4 mRNA expression (Fig. 6D). The expression of Sirt1 protein was significantly reduced in AKI model (Fig. 6E, F). The inhibition of berberine on intestinal inflammation may be partly attributed to the involvement of the Sirt1-NF-κB-TLR4 inflammatory pathway. Berberine downregulated LPS expression (Fig. 6G) in rats with AKI. Berberine attenuates oxidative stress in rats with AKI by downregulating the expression of total SOD (Fig. 6H) in the intestine of AKI. This finding was consistent with the previous study on the effects of berberine on intestinal inflammation [23].

## Discussion

This study evaluated the effect of berberine on changes in the intestinal microbiota and intestinal inflammation induced by renal ischemia‒reperfusion injury in rats with AKI. The main findings of this study are as follows: (1) AKI caused changes in the composition of the intestinal microbiota in rats and induced intestinal microinflammation. It is speculated that intestinal inflammation is also an important factor in promoting the progression of AKI. (2) Berberine can change the intestinal microbiota of rats with AKI, and the protective effect of berberine on the kidneys may be achieved by changing the composition of the intestinal microbiota and inhibiting the intestinal microinflammatory state partly.

Various conditions, such as body’s inflammatory state and oxidative-stress-related organ dysfunction, can lead to enhanced intestinal permeability, which is called “intestinal leakage” [24, 25]. Bacteroidetes and Proteobacteria are the main microbiotas that produce LPS. The endotoxin LPS is produced by Gram-negative bacteria [16]. Among the bacteria that contribute to LPS biosynthesis are *Bacteroidetes*, a type of gram-negative bacteria [16]. Due to intestinal leakage after AKI, intestinal LPS production is increased, leading to increased LPS in the circulation.

When intestinal Sirt1 is pharmacologically activated, TNF-α-mediated intestinal barrier dysfunction, inflammation, and dysregulation are reduced [26]. Sirt1 can regulate intestinal inflammation and susceptibility to IBD by regulating intestinal microbiota [27]. In our experiments, Sirt1 was confirmed to be involved in intestinal inflammation and oxidative stress, and berberine was involved in the Sirt1/NF-κB pathway to achieve intestinal protection.

Berberine promotes the growth of *R. gnavus* among *Lachnospiraceae* family, thereby reducing the development of colonic inflammation in rats with AKI. In this way, the effect of berberine on reducing inflammation in the colon was accompanied by changes in the microbiota, involving a significant increase in the abundance of *Lachnospriaceae* family.

This provides new potential therapeutic options involving berberine [9]. Animal studies have confirmed the protective effect of berberine on renal tubules [28]. However, the studies did not include the observations of changes in the microbiota. Bacteria are generally considered key factors in the progression of AKI, and their role in the etiology of AKI is under discussion.

In our study, *R. gnavus* grew of AKI model of rats, along with the reduction in colonic inflammation caused by berberine. In contrast to this conclusion, studies and meta-analyses have reported that *R. gnavus* can cause several clinical diseases, including newborn allergy disease and Crohn’s disease [29, 30]. However, it is possible that changes in the relative abundances of *R. gnavus* as detected by 16 S rRNA sequencing do not correspond to the changes in absolute numbers but rather reflect the relative repression of other bacterial taxa. A careful analysis is required for these findings. On the one hand, *R. gnavus* may merely be a bystander to mucus-degrading bacteria, profiting from mucus alterations under inflammatory conditions, such as increased mucus thickness and upregulation of Muc2 secretion.

Colon-protective effects were observed in AKI in the berberine group, and berberine supplementation may allow *R. gnavus* to contribute to the microbial environment, thereby limiting bacterial colonization that triggers a strong inflammatory response.

Localized epithelium degradation caused by submucosal bacterial invasion (seen in the models of AKI) results in immune cell activation. It released proinflammatory cytokines including TNF-α and IL-6 and finally enhances leukocyte infiltration and further epithelial cell damage [31]. It has reported that *Bacteroides*, particularly *B. vulgatus*, was more effective than *R. gnavus* at activating dendritic cells and secreting these cytokines [32]. It is probable that BE group will have this effect such as it causes the microbial population to have less *Bacteroides*, which lowers the inflammatory response. Moreover, a severe reduction in the abundance of *Bacteroides* may be the mechanism that provides protection against colonic inflammation in rats with AKI. Our study provided direct evidence for this mechanism because the differences between groups in terms of *Bacteroides* abundance reached a significant level, and *Bacteroides* was also the main marker of differences between groups as revealed by random forest plot analysis.

## Conclusion

In summary, these results reveal the therapeutic benefits of berberine on inflammation-related intestinal injury, altered intestinal permeability and microbiota in AKI models. Despite the promising results obtained in this study, further studies in humans should be conducted to fully understand the onset and progression of AKI, as well as to assess its important role in different stages of AKI. In addition, it would be interesting to follow the evolution of berberine in the development of human AKI through new clinical studies.

### Supplementary Information

**Additional file 1: Figure Supplement 1. **Unedited and labeled Western blot in Fig. 6A.

## Data Availability

The datasets presented in this study can be found in online repositories. The names of the repository or repositories and accession number(s) can be found below: https://www.ncbi.nlm.nih.gov/sra/PRJNA980628. Copyright permission of KEGG pathway maps, etc. in academic publications may be obtained by using the copyright permission request form.
