# Supplementary figures and images for: Berberine alleviates ischemia reperfusion injury induced AKI by regulation of intestinal microbiota and reducing intestinal inflammation
Source: BMC Complement Med Ther. 2024 Jan 30;24:66. doi: 10.1186/s12906-023-04323-y (PMC10826000; doi:10.1186/s12906-023-04323-y)

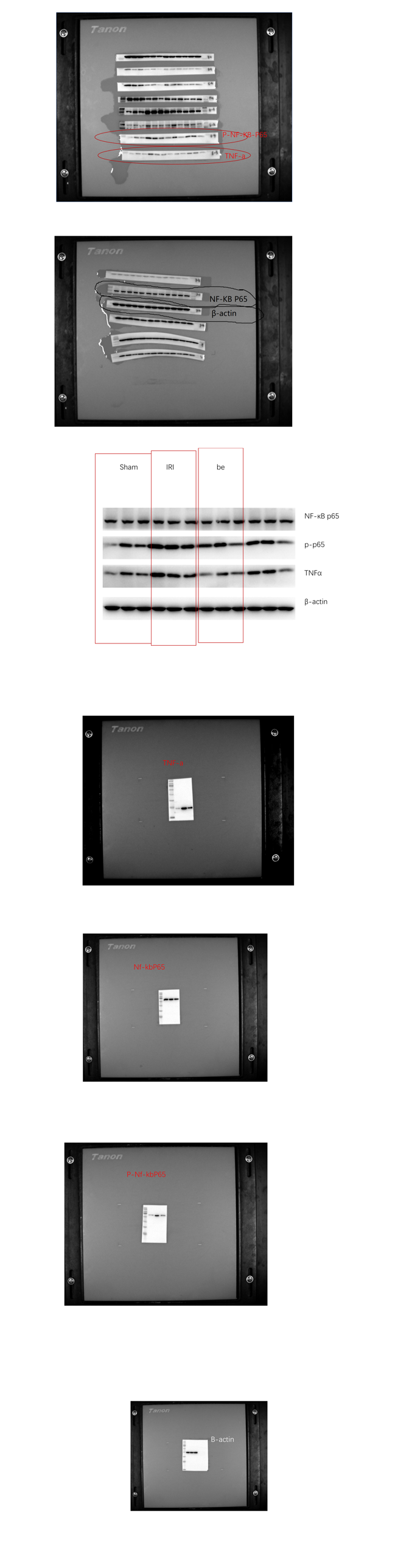

Supplement: Supplementary file 1 — Additional file 1: Figure Supplement 1. Unedited and labeled Western blot in Fig. 6A. [file 12906_2023_4323_MOESM1_ESM.jpg]
